# Multivariate analysis of the phytochemical composition and antioxidant properties in twenty-five accessions across three *Achillea* species

**DOI:** 10.1038/s41598-024-62834-1

**Published:** 2024-05-23

**Authors:** Mostafa Farajpour, Mohsen Ebrahimi, Mohammad Sadat-Hosseini, Dhia Falih Al-Fekaiki, Amin Baghizadeh

**Affiliations:** 1 Crop and Horticultural Science Research Department, Mazandaran Agricultural and Natural Resources Research and Education Center, Agricultural Research, Education and Extension Organization (AREEO), Sari, Iran; 2 Department of Agronomy and Plant Breeding, College of Abourihan, University of Tehran, Tehran, Iran; 3 Department of Horticultural Science, Faculty of Agriculture, University of Jiroft, Jiroft, Iran; 4 Department of Food Sciences, Agriculture College, Basrah University, 61004 Basrah, Iraq; 5 Department of Biotechnology, Institute of Science and High Technology and Environmental Sciences, Graduate University of Advanced Technology, Kerman, Iran

**Keywords:** *A. tenuifolia*, *A. vermicularis*, *A. wilhelmsii*, Total phenol, Ecology, Chemistry

## Abstract

This study explored the chemical composition, antioxidant activity, and total phenol content of aerial parts from 25 accessions of three *Achillea* species (*Achillea wilhelmsii* C. Koch, *Achillea vermicularis* Trin., and *Achillea tenuifolia* Lam.). The plants were collected from various natural habitats across Iran, encompassing regions such as Central, Western, Southern, Northern, Western, and Northwestern parts of the country. Subsequently, they were grown together under field conditions. The study revealed significant variation in essential oil yields among accessions of *A. wilhelmsii*, ranging from 0.01 to 0.107%, *A. vermicularis* with a range of 0.075 to 1.5%, and *A. tenuifolia* showing a variation of 0.1 to 2%. The study utilized Gas Chromatography–Mass Spectrometry (GC–MS) analysis, revealing 75, 49, and 75 compounds in the essential oils of *A. wilhelmsii*, *A. tenuifolia*, and *A. vermicularis*, respectively. Major components included camphor, 1,8-cineole, anethole, α-pinene, and phytol in *A. wilhelmsii*, 1,8-cineole, camphor, levo-carvone, and δ-terpinene in *A. vermicularis*, and β-cubebene, elixene, β-sesquiphellandrene, 1,8-cineole, camphor, and δ-terpinene in *A. tenuifolia*. The essential oil compositions of *A. wilhelmsii* and *A. vermicularis* were predominantly characterized by oxygenated monoterpenes, whereas that of *A. tenuifolia* was characterized by sesquiterpenes. Cluster analysis grouped accessions into three clusters, with *A. tenuifolia* forming a distinct group. Principal Component Analysis (PCA) triplot (62.21% of total variance) confirmed these results and provided insights into compound contributions. Furthermore, total phenolic content and antioxidant activity of the accessions of three species were assessed over 2 years. *A. tenuifolia* exhibited the highest levels in both categories, with statistically significant linear regression between antioxidant activity and total phenol content for *A. tenuifolia* and *A. wilhelmsii*. These findings emphasize significant phytochemical diversity within *Achillea* species, positioning them as promising natural sources of antioxidants. Further exploration and selection of specific accessions within each species are crucial for unlocking their medicinal potential and supporting cultivation and conservation efforts.

## Introduction

Medicinal plants have been demonstrated to play an important role in human health and cultures. Substantial research over the past few decades has identified a wide variety of valuable phytochemicals present among different species^1^. Herbal medicines contribute significantly to fields ranging from nutrition to cosmetics to pharmaceuticals^2^. Traditional medical practices involving herbal remedies have been employed for millennia globally for maintaining wellness and managing illness, as seen traditionally in regions such as China, India, Central and South America, and Africa^3^. Currently, herbal therapies still represent the primary healthcare approach for approximately 85% of people worldwide^4^. Frequently used medicinal plants in the Mediterranean basin particularly include species from families like Lamiaceae, Asteraceae, and Apiaceae. Phytochemical analyses have pinpointed compounds within herbal extracts, essential oils, and fruit juices possessing therapeutic properties^5^. An estimated over 50,000 medicinal plant types exist globally, serving as a rich source for drug finding efforts^6^. Conventional medical systems have long relied on medicinal plants to support health and wellbeing^7^, and they continue playing important roles in modern medicine and pharmacology^8^.

The *Achillea* genus, an esteemed repository of medicinal attributes within the Asteraceae family, boasts a diverse collection of over 100 wild species^9^. Its global utilization extends to proven efficacies in treating various ailments, ranging from gastrointestinal disorders and inflammation to wound healing and diuretic applications^10^. Of particular significance is yarrow, an ancient medicinal plant, prompting meticulous consideration of raw material quality during collection and processing. Essential for subsequent chemophenetic investigations is the analysis of the chemical composition of specialized metabolites, attributing Yarrow's pharmacological effectiveness to compounds such as caffeoylquinic acids, flavonoids, and sesquiterpene lactones, contributing to its multifunctional biological activity^11,12^.

*Achillea* species exhibit a spectrum of effects, including immunosuppressive, anti-inflammatory, and antioxidant properties^13^. Further, these plants demonstrate noteworthy wound-healing and antimicrobial effects against various bacteria, along with antitumor effects on different cell lines. The breadth of their effects extends to anti-arrhythmic, anti-thrombotic, vasorelaxant, anti-hyperlipidemic, anti-hypertensive, hepatoprotective, and gastroprotective actions, as well as endocrine effects like anti-diabetic, estrogenic, and anti-spermatogenic properties^13^. A wealth of ethnopharmacological characteristics associated with *Achillea* has been documented, covering an extensive range of medicinal uses, and the essential oils and extracts have been scrutinized, revealing a diverse array of phytochemicals contributing to therapeutic properties^14^.

Crucial in safeguarding lipids from oxidation and offering numerous health benefits is the role of natural antioxidants^15^. These compounds, instrumental in preventing chronic diseases by mitigating oxidative damage caused by reactive oxygen species (ROS), have gained recent popularity as functional and nutraceutical ingredients, providing natural alternatives to synthetic antioxidants in the food industry^16^. Synthesized through shikimate and phenylpropanoid metabolic pathways in plants, phenolic compounds, including flavonoids and phenolcarbonic acids, stand out for their pharmacological activity in yarrow^16,17^. The diverse properties of *Achillea* plant extracts contribute to their antioxidant potential.

Over the past three decades, significant research has delved into essential oils from *Achillea* species, uncovering predominant monoterpene compounds like 1,8-cineole, camphor, borneol, α- and β-pinenes^9,18–20^.

Medicinal plants, recognized as a valuable resource for drug discovery, offer potential new compounds inspiring scientific innovation^21^. Despite historically serving as the primary form of healthcare in developing nations, only a fraction of Iran's rich medicinal plant species, including *Achillea*, have been thoroughly studied for their phytochemical components and antioxidant activity^9^.

Iran's vast territory harbors a diverse array of medicinal and aromatic plants, including nineteen identified *Achillea* species, seven of which are endemic^22,23^. Despite a wealth of published reports on these plant species, there remains a dearth of information focusing specifically on *Achillea* species in different regions^9,18–20,24^.

*Achillea wilhelmsii* C.Koch is a perennial herb belonging to the Asteraceae family. It grows to a height of 15–30 cm with herbaceous stems and white, petioless leaves. The plant flowers from May to June, producing yellowish-white blooms. It is native to Western Asia and naturally found in Iran. Locally, *A. wilhelmsii* is used traditionally to treat abdominal pain, stomach ache, vomiting, leucorrhoea, dysmenorrhea, stomachache, diabetes, and obesity through decoction and infusion remedies^25^. The main bioactive compounds identified in this plant are carvacrol, linalool, camphor, 1,8-cineole, borneol, and α-pinene^26,27^.

*Achillea tenuifolia* Lam is a native perennial herb that grows to a height of 20–90 cm, with elongated, narrow leaves lacking petioles^28^. It is distributed across Western Asia, Eastern Europe, and the Mediterranean region. The main compounds isolated from this plant are germacrene D, α-humulene and 1,8-cineole^28^. In Iranian traditional medicine, *A. tenuifolia* is implicated as appetite enhancers^29^.

*Achillea vermicularis* Trin. is a perennial herb with several branched stems emerging from the base. It reaches 20–50 cm in height and flowers from late spring through midsummer^30^. Traditionally, indigenous peoples have prepared *A. vermicularis* remedies to cure cold, flu and upset stomach^31^. Previous pharmacological studies have demonstrated these species possesses antidiabetic, antispasmodic, antianxiety, anti-inflammatory, analgesic, and antibacterial properties^26,32–34^.

The climatic diversity in Iran presents an ideal environment for a rich germplasm of medicinal plants, with exciting prospects for discovering unique species with valuable essential oil compositions. These findings hold great promise for advancing human health and well-being through potential therapeutic applications of these plants.

In light of the above, this study aims to bridge the existing gap by evaluating the phytochemical composition, antioxidant activity, and total phenol content of plant extracts from 25 accessions of three specific *Achillea* species: *A. vermicularis*, *A. wilhelmsii* and *A. tenuifolia*. Through a comprehensive analysis, this research seeks to contribute valuable insights into the potential phytochemical components, antioxidant properties, and total phenolic content of these *Achillea* species, thereby enhancing our understanding of their medicinal properties. This knowledge may pave the way for potential therapeutic applications and advancements in human health and well-being.

## Material and methods

### Plant materials

The study gathered seeds from 25 different accessions of three *Achillea* plant species (*A. vermicularis*, *A. wilhelmsii*, and *A. tenuifolia*) in Iran. The voucher samples are stored at the Herbarium of the Research Institute of Forests and Rangelands in Tehran (Table 1). The plants were identified based on Flora Iranica^35^, Identification was confirmed by Dr. Valiolah Mozafarian of the Research Institute of Forests and Rangelands in Iran.

**Table 1:** Geographical location of 25 Iranian *Achillea* sp accessions.

*Achillea* sp.	Code	Voucher numbers	Province	City
*A. wilhelmsii*	W1	8451	Isfahan	Daran
	W2	15796	Lorestan	Kuhdasht
	W3	17628	Qom	Dastjerd
	W4	19489	Kurdistan	Baneh
	W5	33976	Yazd	Tabas
	W6	34431	Hormozgan	Bandar-Abbas
	W7	35561	Mazandaran	Polur
	W8	39346	Qazvin	Tarom Sofla
*A. vermicularis*	V1	9687	Kurdistan	Sanandaj
	V2	9872	Kurdistan	Baneh
	V3	10342	Yazd	Khatam
	V4	19471	West Azerbaijan	Mahabad
	V5	19488	West Azerbaijan	Mirabad
	V6	22593	Kurdistan	Saqqez
	V7	23155	Zanjan	Zanjan
	V8	26032	Kurdistan	Divandarreh
	V9	35179	West Azerbaijan	Khoy
	V10	35181	West Azerbaijan	Salmas
*A. tenuifolia*	T1	14234	West Azerbaijan	Salmas
	T2	14300	Kurdistan	Divandarreh
	T3	25948	Kurdistan	Dehgolan
	T4	25977	Kurdistan	Saqqez
	T5	35180	West Azerbaijan	Mahabad
	T6	39374	Qazvin	Takestan
	T7	34662	Kurdistan	Sanandaj

Initially, the seeds were cultivated in a greenhouse and then transferred to the field when they reached a height of around 10 cm. The seedlings were grown using a randomized complete block design at the research farm of the College of Abouraihan, University of Tehran. Each accession was planted in 1 m^2^ plots with sandy-loam soil. The plants were harvested during the initial flowering stage to assess their phytochemical components, total phenol, and antioxidant activity.

The Pakdasht region, where the study was conducted, experiences distinctive seasonal characteristics. Summers are characterized by extremely hot temperatures, arid conditions, and clear skies, while winters are marked by very cold temperatures, dry air, and mostly clear weather. Throughout the year, temperatures typically range from 1 to 38 °C, with occasional instances of temperatures dropping below − 3 °C or rising above 41 °C. All methods in the study were conducted in accordance with the applicable guidelines and regulations.

### Extracting essential oils

One hundred grams of dried samples from the aerial parts of the plant were samples were ground into a fine powder and then were subjected to hydrodistillation using a Clevenger apparatus for a duration of 3 h.

### Phytochemicals composition of the essential oils

GC–MS analysis was performed using a Varian CP-3800 instrument equipped with a VF-5 capillary column (30 m × 0.25 mm i.d., film thickness 0.25 µm). Helium was used as the carrier gas at a flow rate of 1 mL/min, and the temperature program was set at 60 °C for 1 min, followed by an increase to 250 °C at a rate of 3 °C/min, and held for 10 min. The injector and detector temperatures were maintained at 250 °C and 280 °C, respectively. To identify the components of the essential oils, the retention index (RI) was utilized by subjecting n-alkanes (C6–C24) to programmed temperature conditions. The resulting RI values were then compared to the internal reference MS library (Wiley 7.0) and published data in the literature^36^.

### Extracting the plant extracts

The plant materials were dried at room temperature to remove moisture. Once fully dried, the plants were ground into a fine powder using a mill. For extraction, 5 g of each powdered sample was accurately weighed and transferred to separate Erlenmeyer flasks. To each flask, 50 mL of 80% methanol solvent was added. Extraction was carried out using maceration, where the plant powder was soaked and agitated in the methanol. A magnetic stir plate and orbital shaker set to 150 rpm were used to gently mix the samples at 25 °C for 24 h. After maceration, the mixtures were strained through filter paper to separate the extracts from insoluble residues. The filtered extracts were concentrated by evaporating the methanol under reduced pressure using a rotary evaporator. Then, the pure extract was collected in a small container and stored at 4 °C until total phenol and antioxidant activity analyses. Prior to the analyses, the samples were dried and used immediately.

### Total phenolic content (TPC)

One millilitre of diluted extract (0.1 g in 10 mL of distilled water) was combined with 1 ml of 6 M HCl and 5 mL of 75% methanol/water solution. The resulting mixture was subjected to shaking for 2 h at 90 °C in a water bath. Subsequently, the solution was diluted to a final volume of 10 ml using distilled water. One milliliter of this diluted solution was mixed with 5 ml of previously tenfold diluted Folin & Ciocalteau reagent and 15 ml of sodium carbonate solution (7 g/100 mL). The resulting mixture was brought to a final volume of 100 mL with distilled water. The absorbance of the solution at 760 nm was measured using a spectrophotometer, comparing it against a blank prepared using distilled water instead of the extract, which had undergone the same extraction steps. The experiment was conducted in triplicate, and our methodology closely followed the approach described by Çam et al.^37^, with the exception that we employed four different concentrations of gallic acid solution (1.0, 0.4, 1.6, and 2.2 mg per milliliter) in this study. Finally, the total phenolic content in the extract was quantified and reported as milligrams of gallic acid per milliliter of the sample extract.

### Antioxidant activity

The antioxidant activity of the extract was assessed following the methodology of Brand-Williams et al.^38^, with minor modifications. The experiment employed four different concentrations including 10, 100, 250, and 500 ppm of the extract (0.1, 1, 2.5, and 5 mg in 10 mL of distilled water, respectively). Subsequently, 0.1 ml of each concentration was added to 3.9 ml of a 6 × 10^–5^ mol/L methanol DPPH solution. For the control sample, 0.1 ml of methanol were mixed with 3.9 ml of the methanolic DPPH solution. The spectrophotometer was calibrated using pure methanol as the zero reference. After an incubation period of 30 min, the absorbance of all samples was measured at a wavelength of 515 nm.

### Statistical analysis

The PCA analysis was conducted using version 9.1 of the Statistical Analysis Software (SAS Institute, Cary, NC) for Windows. A heat map clustering analysis was performed to visualize the similarity patterns among samples based on their phytochemicals components values. Hierarchical clustering was applied using Euclidean distance measure and the arithmetic mean method (UPGMA). The heat map displays the clustering of accessions on the y-axis and phytochemicals components on the x-axis, with color intensity indicating the standardized value for each trait in each accession. This analysis helped group accessions exhibiting similar response patterns. Also, a correlation heat map was generated to examine relationships between phytochemicals components. Pairwise correlation coefficients between the components were computed and plotted in a color-coded matrix, with red indicating positive correlation and blue representing negative correlation. The correlation coefficient values were depicted based on their absolute strengths. The results of the clustering, heat map correlation, and triplot analyses were visualized as a colored heat map using MetaboAnalyst^39^. Additionally, the graphs were created using Prism 9 (GraphPad).

## Results and discussion

### Essential oil yield

**Figure 1:**
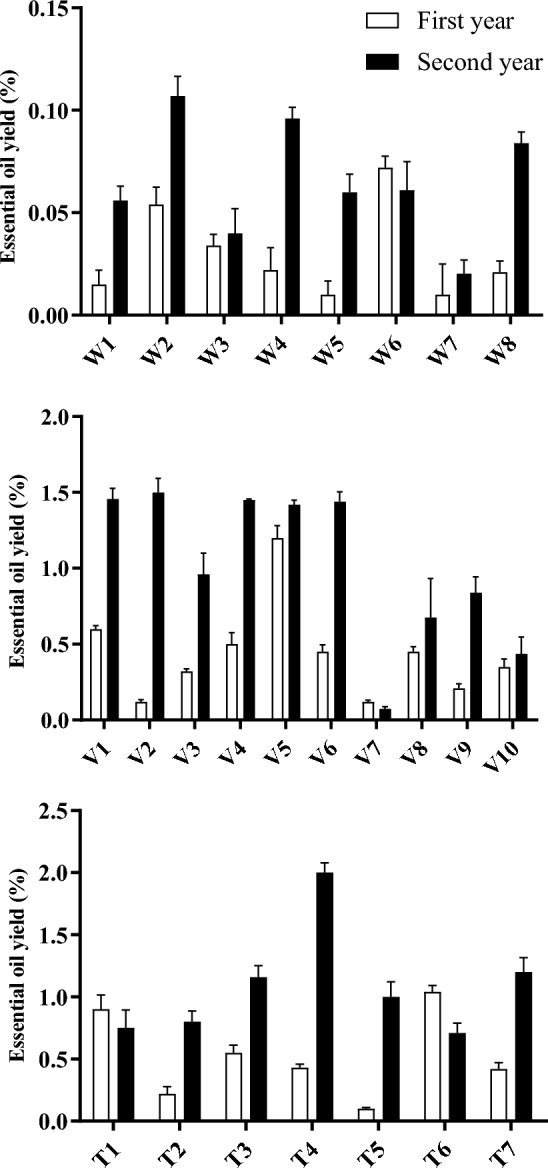
Essential oil yield of 25 accessions of the three studied *Achillea* sp.

The essential oil yield of 25 accessions from three *Achillea* species (*A. wilhelmsii*, *A. tenuifolia*, and *A. vermicularis*) was evaluated over two consecutive years. Figure 1 presents the essential oil yields for each accession during the first and second year of cultivation. In the first year, yields ranged from 0.01 to 1.2% whereas in the second year yields were generally higher between 0.02 and 2%. Statistically significant differences were observed between the 2 years for all three species (*p* < 0.01). Yields increased for the majority of accessions in the second compared to the first year. Certain *A. tenuifolia* accessions such as T4 exhibited notably higher essential oil production in the second year. Based on the ranges observed, *A. tenuifolia* accessions generally exhibited the highest essential oil yields, followed by *A. vermicularis*, with *A. wilhelmsii* having the lowest yields. In the first year, *A. tenuifolia* accessions produced 0.1–1.04% oil, *A. vermicularis* yields varied from 0.12 to 1.2%, while *A. wilhelmsii* yields were under 0.1%. Similarly, in the second year, *A. tenuifolia* accessions yielded 0.71–2%, *A. vermicularis* varied from 0.075 to 1.45%, and *A. wilhelmsii* increased but remained low at 0.02–0.107%. The results demonstrate considerable variation in the essential oil yields among the accessions and between the 2 years. In general, it can be observed that the essential oil yields tend to be higher in the second year compared to the first year for all three *Achillea* species. This finding suggests that the plants undergo certain physiological changes that positively influence essential oil production as they mature. One possible explanation for the increased essential oil yield in the second year is the establishment and development of the plants during the first year. As perennial plants, the first year is typically characterized by a longer growth period until flowering, which occurred in August. The extended growth period in the first year may have prioritized vegetative growth over secondary metabolite production, resulting in lower essential oil yields. In contrast, the second year exhibited a shorter growth cycle, with flowering occurring in May. This shorter growth period likely allowed for increased accumulation of phenolic antioxidants in the aerial parts by the time of flowering, leading to higher essential oil yields. The observed increase in essential oil yield in the second year highlights the importance of considering the stage of plant maturity when studying essential oil production in perennial species. It suggests that the developmental stage and growth cycle significantly influence the biosynthesis and accumulation of essential oil constituents.

The results demonstrate considerable variation in essential oil yields among accessions. For *A. wilhelmsii*, the yields ranged from 0.01 to 0.107% in our study, whereas, the literature reports of 0.14–0.82%^27,40,41^. Similarly, Rabbi Angouran^30^ observed 0.7% yield in *A. vermicularis*, comparable to our observed range of 0.075–1.5% across accessions. For *A. tenuifolia*, Sefidkon et al.^28^ reported a range of 0.16–1.59%, encompassing our observed variation between accessions of 0.1–2%. Overall, the literature comparisons validate the substantial intra-specific variability in oil yields observed among our *Achillea* accessions under uniform cultivation.

### Essential oil compounds

A total of 75 compounds were identified in the *A. wilhelmsii* accessions, as presented in Table 2. Among the identified compounds, camphor was determined to be the predominant constituent in this species. The W5 accession exhibited the highest camphor content (31.48%), whereas the remaining seven accessions displayed varying concentrations of this compound (Fig. S1a). The second most significant compound in this species was 1,8-cineole, with concentrations ranging from 4.31% to 18.82%. The W4 accession exhibited the highest proportion of 1,8-cineole and also displayed another notable compound, anethole, at a concentration of 21.63%.

**Table 2:** Chemical composition of essential oils (%) of eight *A. wilhelmsii* accessions.

Name	RI	W1	W2	W3	W4	W5	W6	W7	W8
2-Methylbutyl acetate	894	0.36	0.48	0.22	0	0.49	0.17	0	0
Santolina triene	932	0	0.18	0	0.1	0	0	0	0
α-Pinene	977	6.65	6.71	1.47	2.39	6.69	1.51	0.98	1.51
Camphene	1003	1.42	1.62	0.55	3.26	4.86	0.55	0.57	1.18
β-phellandrene	1042	3.15	1.85	4.04	3.77	1.1	4.19	0.85	1.39
β-Pinene	1048	1.2	0.6	0.24	0.96	1.26	0.23	0.34	0.44
β.-Myrcene	1071	0.34	0.3	0	0.3	0.17	0	0.61	0.53
Perillen	1109	0.54	0.34	0.19	0	0.48	0.18	0	0.16
4-Carene	1115	0.56	1.5	0.41	0.22	0.83	0.42	0	0.14
m-Cymene	1128	1.22	0.68	0.41	0.13	0.6	0.42	0	0.21
1,8-cineole	1140	15.42	5.74	6.07	18.84	5.29	6.46	4.31	9.47
δ-Terpinene	1186	0.5	1.9	0.28	2.43	0.11	0.29	2.43	0.38
***cis***-Sabinenehydrate	1204	0.59	4.23	0.94	1.49	0.17	0.93	1.13	2.21
Artemesia alcohol	1223	0	0	0	0.13	0	0	0.42	0
α-campholenal	1238	0.4	0.15	0.11	0	0	0.16	0	0
Linalool	1256	7.18	2.59	2.3	0.8	5.4	2.35	1.08	2.46
Butanoic acid,2-methyl-, 2-methylbutyl	1261	1.25	0.72	0.38	0.48	0.89	0.43	0.64	0.26
2-Methylbutyl isovalerate	1270	0.49	0.7	0.7	0	0.35	0.8	0	0.19
Thujone	1284	0	4.91	1.22	0	0	1.22	0	0
Chrysanthenone	1292	0	0.42	5.47	4.3	0	5.63	5.64	10.68
α.-Campholenal	1300	0.76	0.51	0	0.1	0	0	0	0
***trans***-Pinocarveol	1323	0.4	1.59	0.97	0.52	0.29	1	0.67	1.17
Camphor	1333	9.45	17.7	5.88	11.2	31.48	6.45	9.99	14.03
3,9-Epoxy-1-p-menthene	1347	3.22	2.65	1.79	0.25	0.79	1.9	0	0
Pinocarvone	1359	1.04	1.72	2.19	1.01	1.02	2.23	1.6	1.15
Borneol	1374	2.64	6.8	1	1.69	1.21	1.06	2.63	2.23
Pinocamphone	1381	0.34	0	0.17	0	0	0.17	0	0
p-Menth-1-en-4-ol	1388	0.43	5.36	0.72	0.57	0.21	0.73	0.71	0.65
α-Terpineol	1411	2.43	1.53	1.84	0.99	0.85	1.78	1.26	1.69
***trans***-Piperitol	1432	0	0.3	3.79	0	0	3.74	0	2.53
***trans***-Carveol	1448	0.21	1.66	0.33	0	0	0.3	0	0.29
Isogeraniol	1464	0.48	0.21	0.36	0	0.19	0.29	0	0
Piperitone	1499	0	0	0	1.98	0	0	0	13.66
verbenyl acetate	1503	0.47	0	1.37	0.99	0.12	1.36	0.87	0
p-Mentha-1,8-dien-3-one, (+)-	1521	0	0	0.13	0.26	0	0.27	0.41	0.51
α.-Cyclogeraniol acetate	1532	0	2.86	0.63	2.23	0	0.63	0	0
(. + /-.)-Lavandulol, acetate	1540	0	0	4.99	0	0	5.08	0	0
Anethole	1545	1.45	3.93	1.32	21.63	15.27	1.33	8.77	0.56
p-Thymol	1553	0.86	0.34	0.2	0	1	0.19	0	0
***cis***-CarvylAcetate	1607	0.94	0.31	1.31	0	0	1.19	0	0
γ-Terpineol	1641	0.24	2.05	0.17	0	0.16	0.18	0	0.32
Geranyl acetate	1668	0.39	0	0	0	0	0	1.07	0.46
5-Isopropenyl-2-methylcyclopent-1	1689	0	0.65	1.76	1.86	0	1.32	2.61	4.35
Caryophyllene	1721	2.38	1.25	1.5	0.15	0.52	1.59	0.39	0
β-cubebene	1798	1.65	0.51	2.1	3.29	1.24	2.11	3.2	3.42
Elixene	1817	2.43	1.03	2.07	0.29	1.08	1.94	0.63	0.53
α.-Farnesene	1828	0	0	0.33	0.15	0	0.19	0	0
β-Cedrene	1836	0.34	0.16	4.11	0	0	4.38	0	0
Hotrienol	1858	0	0	0.21	0	0	0.21	0.48	0
Longipinocarvone	1912	0.65	0.7	0.65	0	0.43	0.58	1.11	0
Spathulenol	1917	0.27	0	0.43	0.29		0.47	1.21	0.58
Caryophyllene oxide	1924	1.73	0.79	2.42	0.17	0.54	2.56	0.61	0
***trans***-Nerolidol	1958	6	0	0.93	2	0.19	0.94	0.52	0
Agarospirol	1967	0	0	0	0.15	0	0	1.08	0
Spathulenol	1971	0.25	0	0	0	0	0	0.95	0.2
Tetracyclo[6.3.2.0(2,5).0(1,8)]tridecan-9-ol, 4,4-dimethyl-	1978	0.92	1	1.94	0	0.31	1.82	0	0
tau-Cadinol	1981	0	0	0	0.18	0	0	1.82	0.42
14-Methyloxacyclotetradecan-2-one	1986	2.03	1.04	0	0	0.68	0	1.07	0.31
β.-Eudesmol	1989	0	0.6	4.74	0.47	0.22	5.14	1.69	0.58
Acetic acid, 1-methyl-3-(2,6,6-trimethylcyclohex-1-enyl)propyl ester	1994	0	0	1.29	0.1	0	1.63	1	0.6
Humulane-1,6-dien-3-ol	2007	0.51	0.23	2.93	0.2	0	3.13	3.74	0.45
9-Ethylbicyclo(3.3.1)nonan-9-ol	2015	0	0	4.15	0.19	0	4.31	0	0.31
Camazulene	2029	0	0	0.4	0.09	0	0.33	0.52	0
Cyclohexylidenecyclohexane	2044	0.12	0	0.39	0.11	0	0.29	0.78	0.34
Andrographolide	2056	0.26	0	0.15	0	0.42	0.15	0.2	0.19
Farnesol, acetate	2065	0.74	0.2	0	0	0	0.34	1.68	0
Cyclohexadecanolide	2071	2.14	1.11	2.09	2.6	1.64	1.84	7.43	4.47
Phthalicacid, methyl octyl ester	2114	0.38	0.22	0	0.27	0.23	0.28	0.61	0.32
Octadec-9-enoic acid	2122	0.29	0.18	0.29	0.34	0.19	0.21	0.96	0.52
Phytol	2131	3.69	2.18	5.66	2.66	2.83	4.75	4.74	3.24
β.-Cholestanol acetate	2149	0.57	0.14	0.53	0.31	0.44	0.42	0.76	0.29
Tetratriacontane	2167	0.23	0	0.27	0.17	0	0.16	0.31	0.31
Eicosane	2212	0.15	0	0.16	0.14	0	0.13	0.5	1.02
Mehp	2224	2.99	0.78	2.31	1.13	1.94	1.88	1.28	2.12
Hexatriacontane	2273	0	0	0	0	0	0	0.43	0
Monoterpenes hydrocarbons	–	31	21.42	13.66	32.4	21.39	14.3	10.09	15.41
Oxygenated monoterpenes	–	35.66	61.03	36.5	49.99	59.4	37	39.57	58.89
Sesquiterpenes	–	6.8	2.95	10.11	3.88	2.84	10.2	4.22	3.95
Oxygenated sesquiterpenes	–	9.81	2.32	13.39	1.56	3.62	14.5	15.62	2.83

The values in the table are percentages of a given constituent in the total oil. The data were sorted based on the retention index (RI) of the components.

The *A. wilhelmsii* accessions contained α-pinene in quantities ranging from 1% to 6.7%. The W8 accession displayed a noteworthy amount of piperitone (13.66%), which was only found in small amounts in the other accessions. All accessions demonstrated similar levels of phytol compounds. Chrysanthenone component was also detected in the essential oil of this species.

Based on the observed variations in compound number and concentration, it can be inferred that there is considerable phytochemical diversity within this species across different regions of the country. The principal compounds identified in the essential oil of the aerial parts of *A. wilhelmsii* in this study were camphor, 1,8-cineole, anethole, α-pinene, and phytol.

Previous investigations have reported similar compounds, such as camphor, 1,8-cineole, and α-pinene, as well as different compounds, including carvacrol, linalool, and borneol^26,27,42^. The previous reports and the findings of this research suggested that camphor and 1,8-cineole are the principal constituents of the essential oil in this species. Nonetheless, different studies have reported different major compounds for this plant. These disparities may be attributed to variations in physiology, environment, geography, genetics, and plant material diversity^43^. In addition, Saeidi et al.^27^ conducted a study to analyze the essential oil composition of twenty *A. wilhelmsii* accessions collected from their natural habitats across southwest Iran. The researchers identified several components, including chrysanthenone, *trans*-carveol, linalool, neoiso-dihydrocarveol acetate, camphor, filifolone, 1,8-cineole, borneol, α-pinene, *trans*-piperitol, (*E*)-caryophyllene, (*E*)-nerolidol, and lavandulyl acetate, which were present abundantly in the essential oil of *A. wilhelmsii* populations. Many of these components were also detected in the accessions studied in the present research. However, certain components such as neoiso-dihydrocarveol acetate, filifolone, and lavandulyl acetate were exclusively identified in the previous study, whereas components like anethole and phytol were specifically identified in the present study. One possible explanation for these differences is that the accessions in the present study were cultivated in specific locations, while Saeidi et al.^27^ collected accessions from their natural habitats. Also, in the present study, a broader range of locations across the country was covered, which may explain some of the differences observed compared to the previous study.

A total of 75 compounds were identified in *A. vermicularis*, similar to *A. wilhelmsii*, as presented in Table 3. Among these compounds, the composition of 1,8-cineole was recognized as the most significant in this species. The V2 accession exhibited the highest percentage of 1,8-cineole at 26.22% (Fig. S1b). All accessions, except V7, contained varying percentages of this compound. The second most important composition was camphor, with a range of 0% to 28%, and the highest percentage was found in the V6 accession. This accession also exhibited a prominent compound of this species, levo-carvone, at a concentration of 15.38%. The species displayed δ-terpinene in concentrations ranging from 0 to 10%. Several unique compounds were found in the accessions of this species. For instance, V1 contained 10% pinocarvone compound, V7 contained 17.16% cyclohexadecanolide compound, and V9 contained 40.54% pulegone compound. All accessions demonstrated similar percentages of mehp composition. Based on the observed changes in each composition and their respective ranges, it can be inferred that there is significant phytochemical diversity within this species.

**Table 3:** Chemical composition of essential oils (%) of ten *A. vermicularis* accessions.

Name	RI	V1	V2	V3	V4	V5	V6	V7	V8	V9	V10
2-Methylbutyl acetate	894	0	0	0.42	0	0.17	0	0	0	0	0
Santolina trien	931	0	0.2	0	0.13	0.12	0	0	0	0	1.23
α-Pinene	977	4.64	3.08	1.49	2.6	2.02	4.23	0	3.85	0.28	5.13
Camphene	1003	0.81	2.54	0.22	0.71	2.72	2.46	0	2.43	0.11	1.3
β-phellandrene	1042	0.49	4.84	3.24	1.4	3.03	3.6	0	4.82	0.41	4.73
β-Pinene	1049	7.89	1.53	0.32	3.23	0.59	1.02	0	0.91	0.61	1.34
β.-Myrcene	1072	0.47	0.39	0.13	0.51	0.31	0.41	0	0.54	0.2	0.51
Yomogi alcohol	1083	0	0.34	0	0.27	0.27	0	0	0	0.16	0
4-Carene	1115	0.11	0.44	0.63	0.47	0.54	0.16	0	1.17	0	0.38
m-Cymene	1128	0	0.16	0.39	0.12	0.15	0.28	0	0.34	0	0.37
1,8-cineole	1140	8.42	26.22	8.1	15.19	16.7	22.11	0	23.61	7.97	23.57
δ-Terpinene	1186	0.22	9.71	0.24	8.93	10.1	0.74	0	1.93	0	0.48
***cis***-Sabinenehydrate	1204	1.03	2.97	0.8	5.69	3.59	1.01	0	1.98	0	1.56
Artemesia alcohol	1223	0	1.81	0	0.65	0.44	0.09	0	0	0	0
( +)-4-Carene	1232	0	0.21	0	0.16	0.17	0	0	0.48	0.12	0
Linalool	1256	6.25	1.14	0.76	2.63	1.49	0.69	0.55	1.25	0	0.67
Butanoic acid,	1261	0	0.4	0	0.32	0.69	0.32	0	0.67	0	0.77
Thujone	1266	1.01	0	2.05	0	0.39	0	0	0	0	0
Chrysanthenone	1292	1	3.11	0	0.26	5.66	0.54	0.64	3.55	0	0.61
***trans***-Pinocarveol	1323	0.43	0.54	22.73	0.43	0.61	0.67	2.68	1.31	0	1.35
Camphor	1332	2.44	13.44	1.93	5.82	4.14	28.08	0	8.82	0	6.81
9-Ethylbicycl(3.3.1)nonan-9-o	1340	0	0	0	0	0	0	0	0	20.65	0
Pinocarvone	1359	10.5	0.78	2.45	0.54	1.02	0.76	0	2.86	0	2.07
Borneol	1375	2.62	2.07	1.49	1.2	3.31	1.34	1.51	9.83	9.84	1.96
p-Menth-1-en-4-ol	1388	0.46	0	0.48	1.71	1.54	0.56	0	4.29	0	0.78
α.-Thujenal	1393	0	1.58	0.35	0	0	0.24	0	0	0	0
Artemisia ketone	1400	0	0.09	0	0	0.19	0.1	0	0.57	0	0
α-Terpineol	1412	6.41	0.14	0.78	6.54	0.99	2.3	1.19	2.89	0	2.66
***trans***-Piperitol	1432	0.21	0	0	0	0	1.16	0	0.25	0	1.21
***trans***-Carveol	1448	0.36	2.19	0	0.3	0.12	0.14	0	0.89	0	0
Artemisia ketone	1472	0	0	0	0.25	0.46	0.21	0	0	0	0
Pulegone	1477	0	0	0	0	0	0	0	0	40.54	0
Levo-carvone	1484	0	0	0.18	0	0.27	15.38	0	0.55	0	0
Piperitone	1499	0.41	0.32	0	1.11	0.81	0	0.61	0	1.01	21.23
verbenyl acetate	1503	0.33	0	0	0	1.28	0	0	0	0	0
α-Citral	1520	0.15	0.2	0	0	0.35	0	0	0	0.17	0
L-bornyl acetate	1542	0.8	0.21	0	0	21.22	0	0	0.73	0.15	0
Anethole	1546	0.28	3.06	37.63	1.1	0	1.8	1.58	0.28	0.28	3.24
***cis***-CarvylAcetate	1608	0	0.26	0	0	0.12	0	0	0.24	0	0
Pulespenone	1616	0	0	0	0	0	0	0	0	4.73	0
5-Allyl-2-methoxyphenol	1635	0	0.13	0	0	0	0.11	0.48	0.36	0	0.31
Aglaiene	1665	0	0.16	0	0	0.17	0	0	0	0	0
Geranyl acetate	1668	0.75	0	0	0.6	0	0.49	0	0.41	0	0
Methyl eugenol	1698	0.22	0.58	0	0.14	0.39	0.28	1.19	0.44	0	0.31
Caryophyllene	1721	2.12	0	0.42	2.17	0	0	0	0	0	0
α.-Himachalene	1790	0.38	0	0	0.43	0	0	0.59	0	0	0
**β-cubebene**	1798	1.65	2.44	0.15	4.21	3.14	1.18	7.9	4.33	0.35	1.67
Elixene	1817	0.39	0.39	1.34	0.62	0.59	0.28	1.01	0.59	0	0.24
γ-Cadinene	1839	0.61	0	0.33	0.5	0	0	0	0	0	0
δ.-Cadinene	1846	0.14	0	0	0.15	0	0	0	0	0	0
Hotrienol	1858	0.3	0	0	0.19	0	0	1.04	0	0	0
8-Hydroxylinalool	1879	0.32	0	0.17	0	0	0	1.3	0.63	0	0.28
β.-Terpineol	1886	0.43	0	0	0.16	0	0	1.4	0.62	0	0
Longipinocarvone	1912	0.63	0	0.71	0.6	0	0	0	0	0	0
Spathulenol	1917	0.81	0.21	0	0.68	0.63	0.22	3.27	0.48	0.12	0.79
Caryophyllene oxide	1924	1.37	0	0.91	1.39	0.21	0	1.47	0	0	0
α.-Santalene	1950	0.61	0.18	0.14	0.68	0.4	0	2.71	0	0	0.43
***trans***-Nerolidol	1959	3.42	0	0	0.52	0	0	5.86	0	0	0
Agarospirol	1968	2.23	0	0	2.38	0	0	0.82	0	0	0.45
tau-Cadinol	1981	4.67	0.24	0.87	3.65	0.19	0.21	1.38	0.39	0	0.23
β.-Eudesmol	1989	4.82	0.45	0.12	3.24	0.47	0.22	3.85	1.02	0	0.59
Acetic acid, trimethylcyclohex-1	1994	0	0.19	0	0.43	0.21	0.12	1.88	0.3	0	0.19
Humulane-1,6-dien-3-ol	2007	5.73	0.21	0	4.34	0.31	0.33	2.52	0.38	0	0.43
Heptadecane	2013	0	0.13	0.1	0.43	0.11	0.14	1.24	0	0	0
Cyclohexylidenecyclohexane	2044	0.52	0	0.05	0.66	0	0	2.28	0.42	0	0.23
Cyclohexadecanolide	2071	0.84	1.48	1.21	1.18	0.72	1.77	17.16	2.03	0	1.9
Eicosane	2084	0.09	0	0	0.1	0	0	0.31	0	0	0
Phthalicacid, methyl octyl ester	2114	0	0.24	0.26	0.11	0.18	0.24	0.86	0	0	0
Octadec-9-enoic acid	2121	0	0.24	0.18	0.13	0.15	0.19	1.31	0	0	0.11
Phytol	2131	1.22	1.01	2.21	2.38	1	0.71	9.81	0.53	0	0.42
β.-Cholestanol acetate	2149	0.14	0.21	0.21	0.14	0	0	1.21	0	0	0
Hexatriacontane	2167	0.11	0.14	0.25	0.13	0	0	1.02	0.2	0	0
Tetratriacontane	2212	0.12	0	0.12	0.19	0	0	0.79	0.2	0	0
Mehp	2224	0.49	1.52	0.79	0.84	0.98	1.86	1.47	1.4	0.38	2.95
Hexatriacontane	2273	0	0	0.07	0.09	0	0	0.5	0	0	0
Monoterpenes hydrocarbons	–	14.63	22.9	6.27	18.14	19.6	12.62	0	16.13	1.73	15.1
Oxygenated monoterpenes	–	36.71	35	71.8	29.64	49.08	56.27	14.17	43.42	56.72	45.82
Sesquiterpenes	–	5.9	3.17	2.38	8.76	4.3	1.46	12.21	4.92	0.35	2.34
Oxygenated sesquiterpenes	–	24.46	1.42	2.61	17.48	2.15	1.21	21.55	3.01	0.12	2.68

The values in the table are percentages of a given constituent in the total oil. The data were sorted based on the retention index (RI) of the components.

In a study by Rabbi-Angourani^30^, the main compositions of the essential oil of *A. vermicularis* were found to be camphor, bornel acetate, and 1 and 8-cineole. This finding aligns with a report on *A. vermicularis* growth in Turkey, which identified camphor and 15-hexadecanolide as the major components of the essential oil^44^. Previous studies conducted on *A. vermicularis* from Iran also reported 1,8-cineole, camphor, and germacrene D as the main components^45,46^. In another study by Rezaei et al.^47^, the major constituents of the essential oil were identified as camphor, borneol, and terpinen-4-ol. In the present study, the most important components identified in the essential oil of the aerial parts of *A. vermicularis* were 1,8-cineole, camphor, δ-terpinene, anethole, borneol, and *trans*-pinocarveol. These findings are consistent with previous studies conducted in Turkey, which also reported camphor and 1,8-cineole as the most important compounds in this species^48^.

The confirmation of these findings across different regions and years suggests that these compounds are consistently produced under various environmental conditions, although the reported percentages may vary.

The essential oil of *A. tenuifolia* species yielded a total of 49 identified compounds, as outlined in Table 4. It is worth noting that the number of compounds obtained in *A. tenuifolia* was significantly lower compared to the other two species investigated. The most prominent compound observed in *A. tenuifolia* was β-cubebene, which was present in all accessions. Among the accessions, T4 exhibited the highest percentage (50.23%) of β-cubebene, while T3 had the lowest percentage (29.79%) (Fig. S1c). Another notable compound in *A. tenuifolia* was elixene, with a composition range of 5.95 to 8.91%. The T6 accession displayed the highest percentage (13.88%) of elixene, along with β-sesquiphellandrene. Additionally, two compounds, 1,8-cineole and camphor, were identified as major compounds in *A. tenuifolia*. These compounds were also found in the other two species, *A. vermicularis* and *A. wilhelmsii*, where they constituted the primary compounds. In contrast to the other two species, *A. tenuifolia* accessions did not exhibit a unique compound, which could be attributed to the close proximity of the sample collection sites or a lower diversity of chemical compounds in this species. Notably, all accessions of *A. tenuifolia* displayed similar percentages of mehp composition. The key essential compounds identified in the aerial parts of *A. tenuifolia* in this study were β-cubebene, elixene, β-sesquiphellandrene, 1,8-cineole, camphor, and δ-terpinene.

**Table 4:** Chemical composition of essential oils (%) of seven *A. tenuifolia* accessions.

Name	RI	T1	T2	T3	T4	T5	T6	T7
α-Pinene	977	1.6	2.55	3.18	4.98	2.27	2.38	4.7
Camphene	1003	0.36	0.39	0.67	0	0.46	0	0.65
β-phellandrene	1042	0.3	0.79	2.45	1.38	1.07	1.16	1.08
β-Pinene	1048	0.19	0.37	0.51	0	0	0	0.54
Yomogi alcohol	1083	0.54	0	0	0	0	0	0.54
m-Cymene	1128	1.12	1.28	1.53	2.07	1.82	0.86	1.7
1,8-cineole	1140	1.45	4.75	5.23	3.04	6.7	5.39	5.31
δ-Terpinene	1186	5.93	3.54	2.31	1.62	2.35	2.69	4.99
***cis***-Sabinenehydrate	1204	0.51	0.57	1.25	0.94	1.31	0	0.29
Artemesia alcohol	1223	0.81	1.5	0.51	0	0	0.69	2.19
Linalool	1256	0.33	0.36	0.85	0	0.51	0	0.27
Chrysanthenone	1291	0.91	2.3	3.85	0	2.7	0	0
***trans***-Pinocarveol	1325	0.44	1.56	0.88	0	1.65	0	0.63
Camphor	1333	2.33	3.32	5.52	3.96	3.73	2.16	2.95
3,9-Epoxy-1-p-menthene	1347	0.96	2	1.84	1.82	1.32	0.97	1.45
Pinocarvone	1359	0	0.41	0.68	0	0	0	0.42
Borneol	1375	0	0.3	0.46	0	0	0	1.04
p-Menth-1-en-4-ol	1388	0.4	0.57	0.95	1.18	1.19	0	0.4
α.-Terpineol	1411	0.48	1.49	1.01	0	1.55	1.05	1.12
***trans***-Piperitol	1433	0.78	3.82	1.17	0	2.92	1.2	0.58
Piperitone	1499	0	1.32	0.56	0	0	0.59	0.38
verbenyl acetate	1503	2.04	0	1.24	0	0.84	0	0
α.-Cyclogeraniol acetate	1531	0	0	1.66	0	0	0.92	0
Anethole	1546	1.85	0.8	1.14	2.1	2.2	1.39	1.71
Bicyclohexyl	1595	0	0.2	0	0	0	0	0.34
Copaene	1665	0.7	0.5	0.44	0	0	0	0.57
β.-Elemen	1683	0.64	0.49	0.41	0	0.58	0.89	0
Caryophyllene	1721	1.82	1.03	0.93	4.76	0.71	0.99	1.92
(***Z***)-.β.-Farnesene	1763	2.2	3.11	2.23	0	2.23	0.57	1.79
**β-cubebene**	1798	44.89	35.13	29.79	50.23	42.88	36.75	35.99
α.-Farnesene	1811	0	0.83	1.23	1.03	0	0	0.55
Elixene	1817	5.95	7.51	7.13	7.18	6.07	8.91	7.19
δ.-Cadinene	1846	0.75	0.65	0.2	0	0	0	0.86
β-Sesquiphellandrene	1851	0	0.81	1.91	6.87	4.38	13.88	0.8
Spathulenol	1917	3.51	3.13	3.3	1.91	2.1	3.68	2.71
Caryophyllene oxide	1924	0.75	0	0	0	0	0	0.59
Ent-Spathulenol	1926	0	0.81	0.92	0	0	0	0
α.-Santalene	1950	0.29	0.53	0.64	0	0	0	0.85
***trans***-Nerolidol	1959	0.23	0.49	0.58	0	0	0.61	0.41
Spathulenol	1973	0.77	0.96	1.03	0	0.64	1.03	0.84
tau-Cadinol	1981	0	0.6	0.58	1.52	0	0.69	0
α.-Cadinol	1989	3.39	0.94	1.81	0.95	0.88	1.05	1.66
Acetic acid, 1-methyl-3-(2,6,6-trimethylcyclohex-1-enyl)propyl ester	1994	0.66	0.45	0.47	0	0	0.73	0.35
Humulane-1,6-dien-3-ol	2007	1.42	2.35	2.3	1.66	2.22	4.06	1.35
Cyclohexylidenecyclohexane	2044	0.72	0.36	0.37	0	0	0	0.59
Enanthone	2068	0	0	0.52	0	1.02	0	1.24
Cyclohexadecanolide	2071	0.77	0.96	0.47	0	0	0	0
Phytol	2132	0	0	0	0	0	1.51	0.49
Mehp	2224	1.26	0.86	0.77	0.82	1.32	1.27	1.53
Monoterpenes hydrocarbons	–	11.49	13.67	15.88	13.09	14.67	12.48	19.51
Oxygenated monoterpenes	–	10.88	18.32	20.07	8.18	18.6	7.08	11.98
Sesquiterpenes	–	57.49	50.91	45.19	70.07	56.85	61.99	50.88
Oxygenated sesquiterpenes	–	10.07	9.28	10.52	6.04	5.84	11.12	7.56

The values in the table are percentages of a given constituent in the total oil. The data were sorted based on the retention index (RI) of the components.

Previous studies have shed light on the significant chemical compounds of this plant. A study conducted on different parts of the plant reported that flower compounds included limonene and α-cadinol, leaf compounds included limonene, α-pinene, caryophyllene oxide, α-gurjunene, bornyl acetate, and δ-cadinene, while stem compounds included limonene, α-pinene, and spathulenol^49^. Aghjani et al.^50^ identified camphor and borneol as the primary chemical compounds in the flowers of this plant. The compounds contribute to the diverse biological activities of the essential oil and methanol extract of *Achillea* species, including antioxidant and antimicrobial properties^44^.

The major components in the essential oil of the three studied *Achillea* species are presented in Table 5. Among the species, *A. tenuifolia* exhibited significantly higher amounts of β-cubebene and elixene compared to the other two species. Interestingly, all three species had similar levels of α-pinene. In terms of specific compounds, *A. wilhelmsii* displayed higher values of camphor, anethole, phytol, and *trans*-nerolidol compared to the other two species. On the other hand, the amount of 1,8-cineole in *A. vermicularis* was approximately double that of *A. wilhelmsii*, and the amount in *A. wilhelmsii* was approximately double that of *A. tenuifolia*. The essential oil components present in four classes (Tables 2–4). The results showed significant variations in the composition of essential oils among the different *Achillea* species and their classes. In *A. wilhelmsii*, the oxygenated monoterpenes are the dominant class, ranging from 35.66 to 61.03% across the eight accessions. The monoterpene hydrocarbons and sesquiterpenes are also present in notable amounts, but in lower proportions compared to the oxygenated monoterpenes. The *A. vermicularis* samples exhibit a more diverse essential oil profile. The oxygenated monoterpenes are still a significant component, ranging from 14.17 to 71.8%. In contrast, the essential oil composition of *A. tenuifolia* was dominated by sesquiterpenes, which account for 45.19–70.07% of the total essential oil components across the seven accessions.

**Table 5:** Mean values of the major essential oil components in the three *Achillea* sp.

Component	*A. wilhelmsii*	*A. vermicularis*	*A. tenuifolia*
Phytol	3.7	1.9	0.3
Elixene	1.25	0.54	7.13
α-pinene	3.5	2.73	3.1
Anethole	6.8	4.9	1.6
Camphor	13.3	7.15	3.42
1,8-cineole	8.95	15.2	4.55
δ-terpinene	1.4	3.23	3.35
β-cubebene	-	2.71	39.4
***trans***-nerolidol	1.32	1.03	0.33
***trans***-pinocarveol	0.82	3.1	0.74
β-sesquiphellandrene	-	-	4.1

### Multivariate analysis

**Figure 2:**
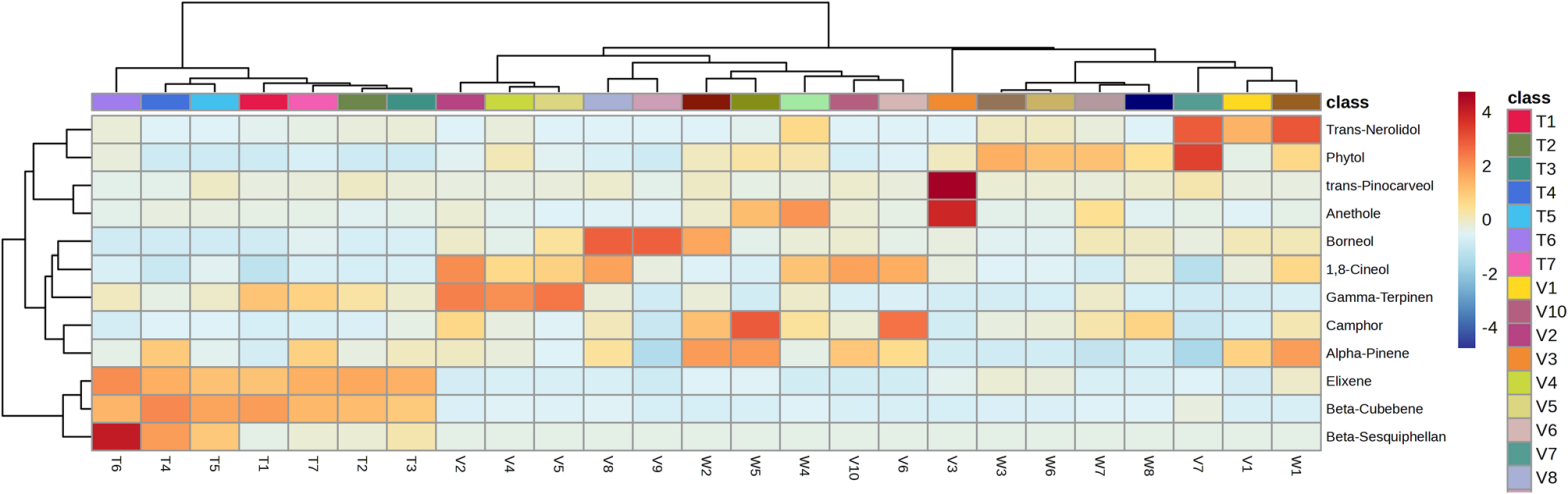
Heat map clustering of the 25 accessions of three studied *Achillea* sp based on the eight measured minerals. The color scales represent the values were normalized by Z-score ((value-mean value)/standard error) for each character.

Cluster analysis was conducted using the key components of essential oils from the studied accessions, namely β-cubebene, elixene, borneol, camphor, 1,8-cineole, α-pinene, δ-terpinene, phytol, anethole, β-sesquiphellandrene, *trans*-pinocarveol, and *trans*-nerolidol. The analysis resulted in the classification of the accessions into three main groups (Fig. 2). The first group comprised seven accessions of *A. tenuifolia*. Based on the results, the accessions of this species were distinguished from other accessions primarily due to significantly higher levels of β-cubebene and elixene components in their essential oils. Within this group, accession T6 was separated from other *A. tenuifolia* accessions due to its high level of β-sesquiphellandrene in its essential oil. The second and third groups were formed by the accessions of *A. wilhelmsii* and *A. vermicularis*. The cluster analysis did not differentiate between the accessions of these two species, indicating a similarity in their essential oil compositions. However, accession V3 did not belong to the second and third groups due to its elevated levels of anethole and *trans*-pinocarveol components in its essential oil.

**Figure 3:**
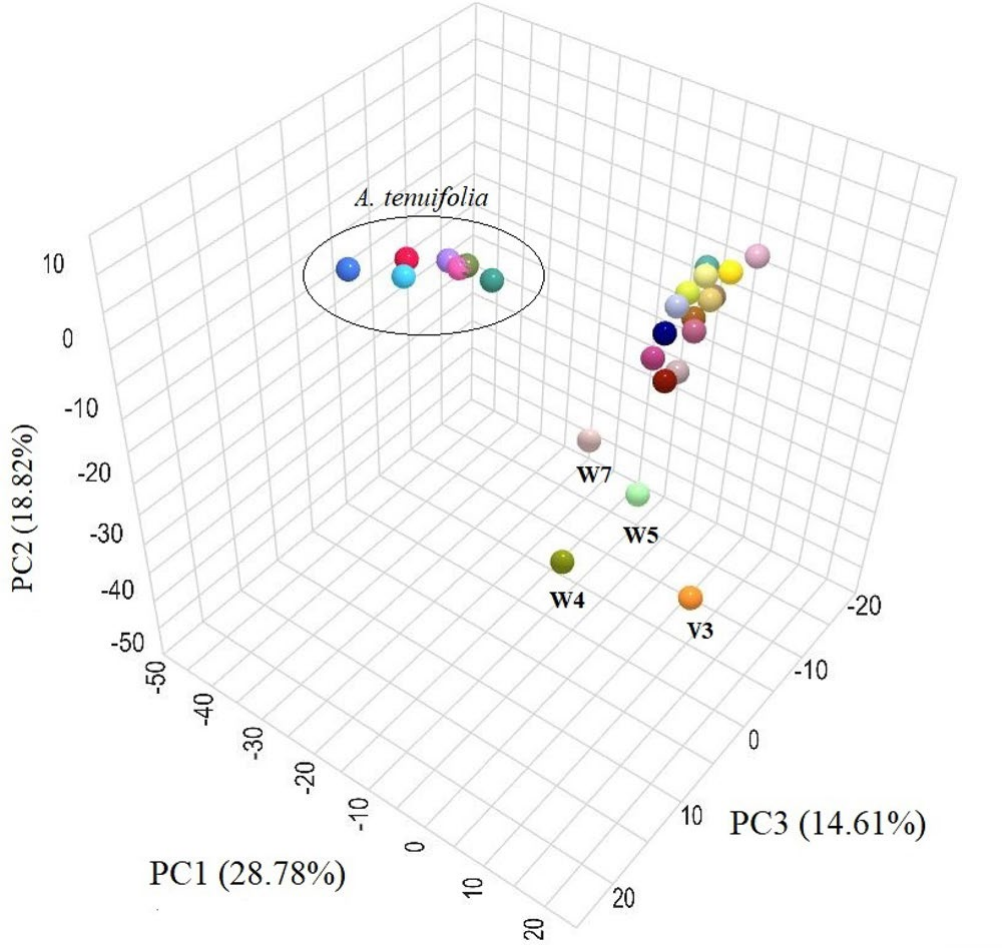
PCA triplot based on the three first PC of the 25 accessions of three studied *Achillea* sp.

The results of principal component analysis (PCA) revealed that the first three principal components (PCs) accounted for 62.21% of the total variance (Table 6). PC1, which explained 28.78% of the variance, exhibited a significant positive correlation with β-cubebene, elixene, and β-sesquiphellandrene, and a significant negative correlation with borneol and camphor. PC2, explaining approximately 18.82% of the variance, showed positive correlations with 1,8-cineole, α-pinene, and δ-terpinene, while displaying negative correlations with phytol and anethole. Additionally, PC3 explained 14.61% of the total variation among the study accessions and was positively correlated with *trans*-pinocarveol, while negatively correlated with *trans*-nerolidol. The PCA triplot confirmed the clustering results, as the accessions of *A. tenuifolia* were closely grouped together (Fig. 3). Also, some accessions from *A. wilhelmsii* and V3 were found to be distant from other accessions of the same species, as well as from *A. vermicularis* accessions.

**Table 6:** PCA based on the eight minerals of 25 *Achillea* sp. accessions.

Label	Minerals	Principal components		
		PC1	PC2	PC3
1	β-cubebene	0.95	0.05	0.05
2	Elixene	0.95	− 0.02	0.05
3	β-sesquiphellandrene	0.73	0.00	0.03
4	Borneol	− 0.52	0.27	− 0.09
5	Camphor	− 0.47	0.41	0.06
6	1,8-cineole	− 0.52	0.59	0.13
7	α-pinene	− 0.09	0.51	− 0.03
8	δ-terpinene	0.20	0.46	0.10
9	Phytol	− 0.37	− 0.70	− 0.44
10	Anethole	− 0.30	− 0.43	0.76
11	*Trans*-pinocarveol	− 0.19	− 0.53	0.75
12	*Trans*-nerolidol	− 0.18	− 0.51	− 0.61
–	Eigenvalue	3.45	2.25	1.75
–	% of variance	28.78	18.82	14.61
–	Cumulative%	28.78	47.60	62.21

Cluster analysis and PCA have played a pivotal role in advancing our understanding of the chemical compositions of essential oils derived from different *Achillea* species. Yener^51^ employed PCA to successfully identify *A. nobilis* subsp. neilreichii as distinct in terms of its composition, while Turkmenoglu^44^ utilized PCA to group species based on their chemotypes. These studies exemplify the effectiveness of PCA in discerning unique chemical profiles within the *Achillea* genus. Similarly, Sadyrbekov^52^ employed cluster analysis to categorize species according to their chemical compositions, further underscoring the significance of these analytical techniques in comprehending the diverse essential oil compositions found in *Achillea* species.

**Figure 4:**
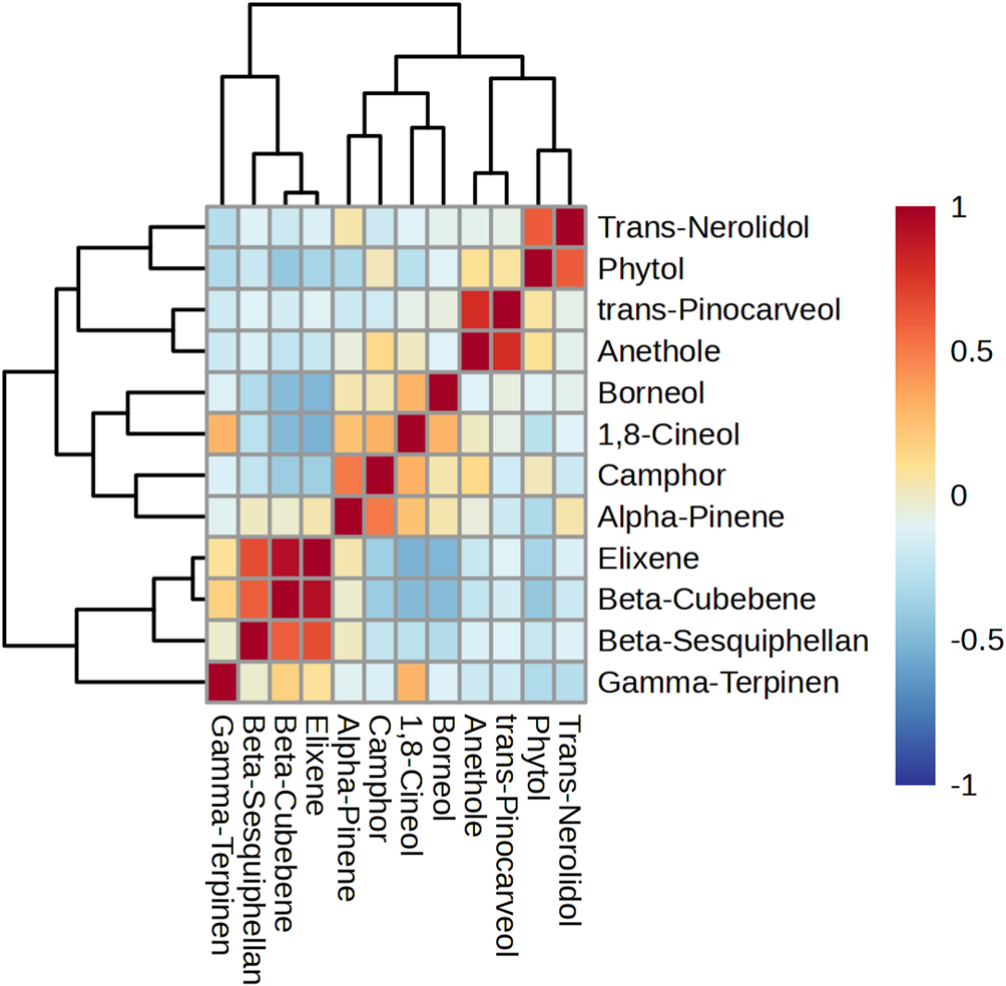
Heat map correlation among the major essential oil composition of 25 accessions of three studied *Achillea* sp.

The correlation coefficients among the top essential oil components of the accessions of three *Achillea* species, including *A. wilhelmsii*, *A. vermicularis*, and *A. tenuifolia*, were presented as heat map correlation (Fig. 4). The results indicated the strength and direction of the relationships between the components. β-sesquiphellandrene exhibited a moderate positive correlation with β-cubebene, elixene, and *trans*-pinocarveol. β-cubebene displayed a strong positive correlation with elixene and a moderate positive correlation with *trans*-pinocarveol. It also had weak negative correlations with α-pinene, camphor, 1,8-cineole, borneol, anethole, phytol, and *trans*-nerolidol. Elixene showed a strong positive correlation with *trans*-pinocarveol. It also had weak negative correlations with α-pinene, camphor, 1,8-cineole, borneol, anethole, phytol, and *trans*-nerolidol. Anethole showed a strong positive correlation with *trans*-pinocarveol and a weak positive correlation with phytol. Phytol showed a moderate positive correlation with *trans*-nerolidol.

These correlation coefficients provide insights into the relationships between the essential oil components and can be used to understand the composition and characteristics of the different *Achillea* species accessions.

### Antioxidant activity and total phenol content

In this study, the total phenol content and antioxidant activity of the samples were assessed over a period of 2 years.

**Figure 5:**
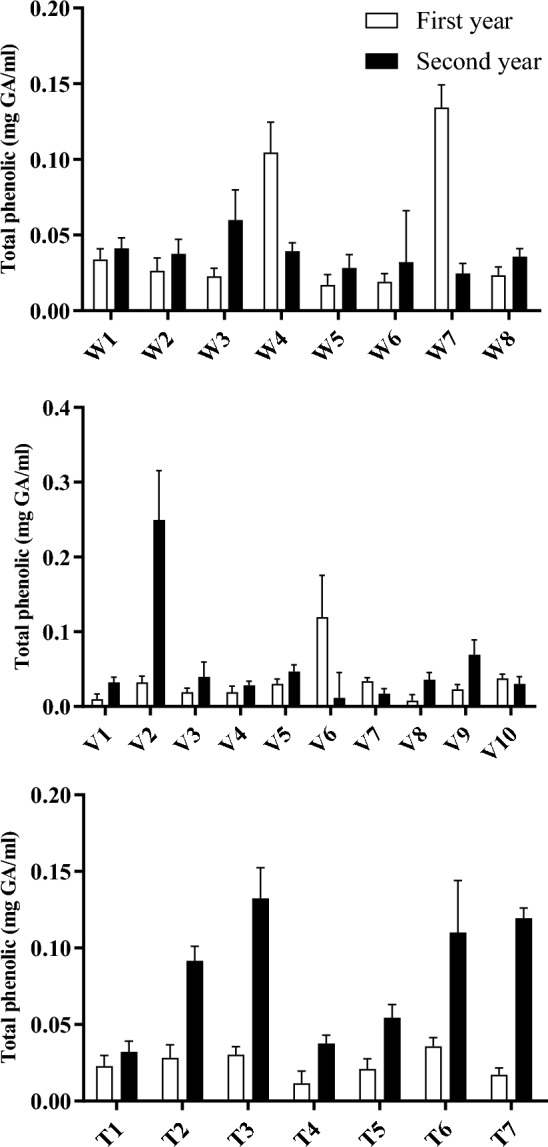
Total phenol contents of 25 accessions of the three studied *Achillea* sp.

The analysis of *A. wilhelmsii* species revealed that the W4 and W7 accessions exhibited higher levels of total phenol content in comparison to other accessions in the initial year (Fig. 5). These two accessions were the sole ones that displayed elevated phenol levels in the first year compared to the second year, while the remaining accessions demonstrated higher phenol levels during the second year relative to the first. A paired t-test was employed to compare the 2 years in terms of this characteristic, which revealed no statistically significant difference between the two periods. Furthermore, the W4 (IC_50_ = 278.32) and W7 (IC_50_ = 243.21) accessions exhibited greater antioxidant activity in the first year when compared to other samples (Table 7), whereas in the second year, the W3 accession (IC_50_ = 203.23) displayed the highest antioxidant activity. With the exception of the W7 accession, all accessions demonstrated higher antioxidant activity in the second year compared to the first. Regression analysis for each accession conducted for each year demonstrated statistically significant models (*p* < 0.01). Moreover, the coefficient of determination (R-squared) exceeded 0.93 in the majority of models, indicating a high degree of accuracy for the models.

**Table 7:** Antioxidant activities of 25 accessions of three studied *Achillea* sp.

Accessions	Year1				Year2			
	IC_50_	b	a	R^2^	IC_50_	b	a	R^2^
W1	333.37	0.14	− 0.5	0.93	252.44	0.121	18.75	0.96
W2	360.91	0.11	10.3	0.96	259.34	0.086	20.33	0.78
W3	343.07	0.13	5.4	0.98	203.23	0.178	9.79	0.94
W4	278.32	0.13	− 0.36	0.99	258.26	0.11	11.1	0.99
W5	387.38	0.17	− 5.58	0.98	320.02	0.182	2.8	0.95
W6	360.71	0.16	− 3.34	0.96	282.63	0.13	15	0.99
W7	243.21	0.15	− 12	0.98	332.12	0.164	8.6	0.99
W8	413.33	0.16	− 1.35	0.99	269.23	0.167	2.8	0.98
V1	373.33	0.15	− 6	0.99	260.77	0.13	16.1	0.97
V2	361.18	0.135	1.24	0.95	212.76	0.17	13.83	0.94
V3	367.46	0.13	2.23	0.94	248.07	0.15	12.79	0.94
V4	369.51	0.124	4.18	0.99	274.17	0.12	17.1	0.97
V5	362.19	0.169	− 11.21	0.99	244.38	0.13	18.23	0.88
V6	290.47	0.148	7.01	0.99	402.50	0.1	9.75	0.61
V7	356.58	0.152	− 4.2	0.98	309.00	0.1	19.1	0.82
V8	390.27	0.146	− 6.98	0.99	255.39	0.18	4.03	1
V9	362.20	0.127	4	0.98	230.00	0.13	20.1	0.8
V10	347.73	0.11	11.75	0.99	260.87	0.16	8.26	0.99
T1	352.96	0.142	− 0.12	0.99	309	0.1	19.1	0.82
T2	345.99	0.142	0.87	0.98	244.92	0.13	18.16	0.89
T3	326.92	0.13	7.5	0.99	216.25	0.16	15.4	0.95
T4	385.95	0.121	3.3	0.98	290	0.1	21	0.84
T5	361.18	0.135	1.24	0.95	282.09	0.11	18.97	0.90
T6	255.60	0.157	9.87	0.99	243.18	0.17	8.66	0.98
T7	364.03	0.149	− 4.24	0.98	225.16	0.19	7.22	0.97
t-value	3.83* (indicating that the antioxidant activity of the 2 years of the study differed significantly at a significance level of 0.05.)							

**Figure 6:**
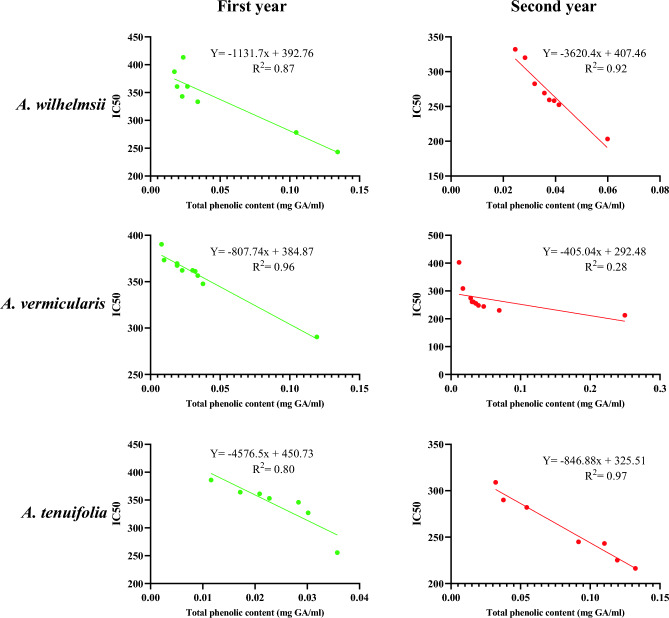
The relationships between antioxidant activity and total phenol content of three *Achillea* species over 2 years.

Although there was no significant difference observed in total phenol content across the 2-year period, there was a noteworthy difference in antioxidant activity (*p* < 0.05). Figure 6 illustrates the relationship between antioxidant activity and total phenol content. Linear regression analysis for these two variables was statistically significant, and the coefficient of determination was relatively high for both years within this species. While phenolic compounds are widely recognized as the principal bioactive compounds associated with antioxidants^16^, it should be noted that total phenol content does not encompass the entirety of antioxidants^53^.

The analysis of *A. vermicularis* species revealed that the V2 accession exhibited a significantly higher total phenol content in the second year compared to other accessions. Conversely, the V6 accession displayed the highest phenol levels in the first year. Apart from these two accessions, there were no significant differences in phenol content among the accessions over the 2-year period. Notably, the V2 and V6 accessions, characterized by higher phenol levels, also demonstrated superior antioxidant activity compared to other samples. In the second year, all accessions, except for V6, exhibited higher antioxidant activity relative to the first year. The regression analysis results for each accession in each year established the statistical significance of the obtained models (*p* < 0.01), with the exception of the model associated with the V6 accession in the second year. Furthermore, the coefficient of determination yielded high values in most cases, indicating a strong model accuracy. Although there was no significant variation in total phenol content throughout the 2-year period, antioxidant activity displayed a significant difference (*p* < 0.01). Linear regression analysis confirmed the significance of this relationship for the two variables in the first year, supporting the linearity of the model. However, in the second year, the relationship between the variables was found to be non-linear.

The analysis of *A. tenuifolia* species revealed that the highest total phenol contents in the first and second years were obtained from T6 and T3 accessions, respectively. The range of variation in total phenol content was low in the first year but increased in the second year. The t-test analysis indicated a significant difference between the 2 years for both total phenol content and antioxidant activity (*p* < 0.01). All accessions demonstrated higher antioxidant activity in the second year compared to the first year. The results of regression analysis for each accession in each year revealed the statistical significance of the obtained models (*p* < 0.01). Additionally, the coefficient of determination exhibited high values in most cases. The results of linear regression analysis revealed a linear relationship between antioxidant activity and total phenol content in both years.

The results demonstrated that among the examined species, *A. tenuifolia* displayed the highest level of antioxidant activity. However, there was a relatively comparable level of antioxidant activity observed across the studied species. Additionally, *A. tenuifolia* exhibited the highest total phenol content.

The year factor had a significant effect on the antioxidant activity of all three studied *Achillea* species, while it was only statistically significant for total phenol content in *A. tenuifolia*. As the plants are perennial, in the first year of establishment, the growth period until flowering was longer compared to the second year. In the first year, the plants flowered in August, while in the second year flowering occurred in May. The extended growth period in the first year likely diverted more resources towards vegetative growth rather than secondary metabolite production^54^. In contrast, the shorter growth cycle in the second year allowed for increased accumulation of phenolic antioxidants in the aerial parts by the time of flowering in May. This may explain the higher antioxidant activity levels observed in all three species during the second year. Meanwhile, the year effect on total phenol content was only significant for *A. tenuifolia* possibly due to greater sensitivity or capacity for phenolic accumulation in this species.

The results of present study are consistent with a study conducted by Polatoglu et al.^48^, which reported significant DPPH scavenging activity in the essential oils of *A. tenuifolia* and *A. vermicularis*. Several *Achillea* species, such as *A. vermicularis*, *A. wilhelmsii*, and *A. tenuifolia*, have been identified as possessing noteworthy antioxidant activity and exhibiting high total phenolic content^45,55–57^. These properties can be attributed to the presence of bioactive compounds, including phenolics and essential oils, in these species^56,58^. However, it should be noted that the antioxidant activity of *A. tenuifolia*'s root extracts does not necessarily correlate with their total phenol content^57^. Also, Al-Ogaili et al.^59^ revealed that Iraqi *A. tenuifolia* contains high levels of polyphenols, indicating its potential as a source of antioxidants. These findings underscore the potential of *Achillea* species, including *A. vermicularis*, *A. wilhelmsii*, and *A. tenuifolia*, as natural sources of antioxidants with promising applications in the pharmaceutical and medical fields. Also, significant antioxidant properties and total phenol content were observed in *Achillea* species collected from their original site^56^. These plants exhibited higher antioxidant properties and total phenol content compared to our study, which may be attributed to different factors such as elevation, region, and organs used^60–62^.

## Conclusion

To highlight the novel findings of this study, the results revealed substantial differences in the essential oil profiles and antioxidant potentials among accessions from three *Achillea* species (*A. wilhelmsii*, *A. tenuifolia*, and *A. vermicularis*) when cultivated under uniform field conditions. Notably, evaluating multiple accessions together for the first time demonstrated considerable intraspecific chemical diversity and phenolic variations between genotypes of the three species that had not been previously reported. The dominant compounds differed between the species, with camphor being predominant in *A. wilhelmsii*, 1,8-cineole in *A. vermicularis*, and β-cubebene and elixene in *A. tenuifolia*. However, certain compounds, such as 1,8-cineole and camphor, were consistently found across all species. Cluster analysis grouped the accessions into three main clusters, with *A. tenuifolia* accessions forming a distinct group characterized by higher levels of β-cubebene and elixene. Additionally, the study assessed the total phenolic content and antioxidant activity of *Achillea* species over a 2-year period. Among the examined species, *A. tenuifolia* exhibited the highest levels of total phenol content and antioxidant activity. However, there was a relatively comparable level of antioxidant activity observed across the studied species. Furthermore, linear regression analysis revealed a positive relationship between antioxidant activity and total phenol content in both years for *A. tenuifolia* and *A. wilhelmsii*. These findings emphasize the phytochemical diversity within *Achillea* species and highlight the influence of genetic and environmental factors on their essential oil compositions and antioxidant properties. Moreover, the study underscores the potential of *Achillea* species as a reliable source of antioxidants for use in the food and pharmaceutical industries. Further research, including the selection of specific accessions within each species, can provide deeper insights into their chemical composition and medicinal potential.

## Data Availability

All data are within the manuscript.
